# Multicenter machine learning study for long-term prediction of acute kidney injury after complete mesocolic excision: integrating inflammatory biomarkers and transfusion-related risk factors

**DOI:** 10.3389/fonc.2026.1782613

**Published:** 2026-03-26

**Authors:** Shunpeng He, Yilin Wu, Yuan Liu

**Affiliations:** 1 Department of Emergency Medicine, Sir Run Run Shaw Hospital, Zhejiang University School of Medicine, Hangzhou, China; 2 Department of General Surgery, The Affiliated Tengzhou Central People’s Hospital of Xuzhou Medical University, Zao Zhuang, China

**Keywords:** acute kidney injury, complete mesocolic excision, external validation, inflammatory response, machine learning, perioperative blood transfusion, predictive model

## Abstract

**Background:**

Complete mesocolic excision (CME) is a technically complex and highly invasive surgical approach, and patients undergoing CME are consequently at elevated risk of postoperative acute kidney injury (AKI). Despite this vulnerability, reliable tools for individualized AKI risk prediction in this population remain unavailable. Moreover, the contributions of perioperative inflammatory responses and blood transfusion to AKI pathogenesis have not been comprehensively examined. Here, we sought to develop and validate a multicenter, machine learning–based model to predict AKI following CME and to delineate the relative impact of inflammation- and transfusion-related determinants.

**Methods:**

We retrospectively enrolled patients with colon cancer who underwent CME between 2010 and 2020 at five tertiary referral centers. Patients were allocated to an internal cohort or an external validation cohort according to hospital of origin. The internal cohort was randomly divided into training and validation subsets in a 7:3 ratio. Feature selection and model construction were performed using multivariable analyses and five machine learning algorithms: Extreme Gradient Boosting (XGBoost), Random Forest, Support Vector Machine, k-Nearest Neighbors (KNN), and Multilayer Perceptron (MLP). Model performance was assessed using receiver operating characteristic (ROC) curves, calibration plots, decision curve analysis (DCA), and k-fold cross-validation, followed by independent external validation. Model interpretability and the quantitative contribution of inflammatory and transfusion variables were evaluated using SHapley Additive exPlanations (SHAP).

**Results:**

Among the evaluated models, XGBoost achieved the most favorable performance, exhibiting superior discrimination, calibration, clinical utility, and generalizability, with area under the curve (AUC) values of 0.92 in the training set, 0.88 in the validation set, and 0.922 in the external cohort. SHAP analysis highlighted tumor size, operative duration, preoperative anemia, postoperative neutrophil-to-lymphocyte ratio (NLR), intraoperative blood loss, C-reactive protein (CRP), intraoperative hypoxemia, and perioperative blood transfusion as the dominant predictors of AKI. Notably, inflammation-related markers (NLR and CRP) and transfusion-related factors exerted a substantial influence on AKI risk.

**Conclusion:**

We established an interpretable, multicenter machine learning–based model with high predictive accuracy, robustness, and clinical relevance for AKI following CME. Our findings identify perioperative inflammation and blood transfusion as key drivers of postoperative AKI, offering mechanistic insight and a foundation for early risk stratification and targeted preventive strategies in high-risk patients.

## 1 Introduction

Complete mesocolic excision (CME) is widely recognized as a standardized radical surgical strategy for colon cancer. By adhering to the principles of central vascular ligation and anatomically precise mesocolic dissection, CME has been shown to reduce local recurrence and to confer durable improvements in long-term oncological outcomes (1–3). Nonetheless, the extensive operative field, greater depth of dissection, and heightened physiological stress imposed on hemodynamics and tissue perfusion render postoperative morbidity an enduring clinical challenge. Among these complications, acute kidney injury (AKI) is particularly prevalent and clinically consequential after major abdominal surgery (4, 5). AKI is a common and serious clinical syndrome characterized by a rapid decline in renal function over a short period, manifested by elevated serum creatinine, reduced urine output, or both. AKI can result from a variety of prerenal, intrinsic renal, and postrenal factors, with underlying mechanisms often involving intraoperative hypotension and hypovolemia leading to renal hypoperfusion, decreased tissue oxygen delivery, postoperative systemic inflammatory responses, as well as exposure to certain drugs or metabolic disturbances. AKI can prolong hospital stay, increase susceptibility to postoperative infections, substantially raise healthcare costs, and is closely associated with elevated short- and long-term mortality (6–8). Accumulating evidence further indicates that postoperative AKI not only impairs short-term recovery but also precipitates sustained renal dysfunction, including progression to chronic kidney disease or even end-stage renal disease, thereby exerting a long-lasting adverse effect on patients’ quality of life (9–11).

In routine clinical practice, the detection of AKI following CME largely depends on postoperative laboratory surveillance and clinical observation. Renal function is typically evaluated through serial measurements of serum creatinine, urine output, and electrolyte profiles, with postoperative renal impairment generally recognized only after overt increases in creatinine, the onset of oliguria, or manifest disturbances in fluid and electrolyte homeostasis (12–14). Such event-driven and reactive monitoring strategies are intrinsically delayed and frequently fail to capture early renal hypoperfusion or subclinical kidney injury.

Moreover, the anatomical and technical attributes intrinsic to CME—including central vascular ligation, extensive mesocolic resection, intraoperative traction, and the attendant risk of regional hypoperfusion—render renal perfusion particularly vulnerable during the perioperative period (2, 15, 16). Conventional assessment paradigms inadequately account for the multidimensional determinants of AKI risk, encompassing preoperative comorbidities, intraoperative hemodynamic variability, fluid management, blood loss, and transfusion requirements. Consequently, perioperative decision-making often relies on empirical judgment rather than objective, quantitative tools. Although isolated hemodynamic metrics, such as nadir intraoperative mean arterial pressure or urine output, may provide limited prognostic insight, their predictive utility is modest and readily confounded by intravascular volume status, anesthetic depth, and pharmacological interventions.

As a result, traditional perioperative AKI management has been largely diagnostic rather than predictive in orientation. This paradigm constrains the early identification of high-risk individuals before or during surgery and impedes the timely implementation of targeted preventive measures, including goal-directed fluid therapy, individualized blood pressure management, and avoidance of nephrotoxic exposures (17–19). Against this backdrop, the rapid evolution of machine learning (ML) and multivariable risk modeling has highlighted the shortcomings of conventional, single-parameter or experience-driven approaches to AKI assessment (20–22). Unlike traditional statistical techniques, ML algorithms can accommodate high-dimensional, heterogeneous, nonlinear, and complex clinical data, integrating preoperative characteristics, intraoperative hemodynamics, fluid and transfusion strategies, blood loss, and postoperative laboratory indices to generate more precise and individualized risk estimates. By uncovering latent patterns and interactions within the data, ML enables earlier identification of patients at heightened risk while simultaneously quantifying the relative contribution of individual features, thereby aligning interpretability with clinical decision support.

Leveraging comprehensive multicenter clinical data from CME cohorts, the present study aims to construct a machine learning–based model for predicting postoperative AKI risk. By integrating key preoperative and intraoperative variables, we seek to identify patients at elevated risk of AKI after CME and to delineate the relative importance of major contributory factors. This framework is intended to support evidence-based preoperative risk stratification, optimize perioperative management, and facilitate early intervention, ultimately enhancing perioperative safety and improving outcomes for patients undergoing CME.

## 2 Materials and methods

### 2.1 Study subjects

The data analyzed in this study were obtained from the clinical databases of Wuxi People’s Hospital (affiliated with Nanjing Medical University), Wuxi Second People’s Hospital, Tengzhou Central People’s Hospital, Tengzhou Hospital of Traditional Chinese Medicine, and Gaomi People’s Hospital, including patients with colon cancer who received treatment between January 2010 and January 2020. Eligibility criteria included: (1) receipt of laparoscopic-assisted or conventional open CME; (2) age ≥18 years; (3) procedures performed by experienced surgical teams with independent expertise in CME; and (4) postoperative pathological confirmation of colon cancer.

Preoperative renal function was systematically evaluated in all patients in accordance with the Kidney Disease: Improving Global Outcomes (KDIGO) guidelines. All serum creatinine measurements obtained within 7 days before surgery were reviewed, and the earliest stable value was designated as the baseline creatinine. Exclusion criteria were as follows: (1) evidence of preoperative acute kidney injury, defined as an increase in serum creatinine of ≥0.3 mg/dL within 48 hours or to ≥1.5-fold above baseline within 7 days before surgery; (2) advanced chronic kidney disease (stage 4–5; estimated glomerular filtration rate <30 mL/min/1.73 m²), end-stage renal disease, or long-term maintenance hemodialysis or peritoneal dialysis; (3) prior renal transplantation or conditions associated with pronounced renal function variability due to immunosuppressive therapy; (4) requirement for complex concomitant surgical procedures, such as major hepatectomy or urological reconstruction, with the potential to substantially perturb perioperative hemodynamics; (5) extensive missing data, particularly the absence of key perioperative variables precluding robust model development; (6) occurrence of severe postoperative complications within 30 days, including shock, diffuse peritonitis, or postoperative mortality; (7) pregnancy; and (8) concomitant malignancies or evidence of distant metastasis from colon cancer confirmed by pathological or imaging assessments.

All patients were followed for a minimum of 3 years after surgery. This retrospective study was approved by the Ethics Committees of Wuxi People’s Hospital (affiliated with Nanjing Medical University), Wuxi Second People’s Hospital, Tengzhou Central People’s Hospital, Tengzhou Hospital of Traditional Chinese Medicine, and Gaomi People’s Hospital. Written informed consent was obtained from all participants, and all personal identifiers were removed prior to analysis. The ethics approval number was KY22086 and IEC-AF/17-1.1.

### 2.2 Study design and data collection

In this study, perioperative variables were prospectively defined and systematically collected across three temporal phases: preoperative, intraoperative, and postoperative. Preoperative variables were obtained within 24 h before surgery and encompassed demographic characteristics (sex, age, body mass index [BMI], smoking status, and alcohol consumption), baseline clinical parameters (American Society of Anesthesiologists [ASA] classification and Nutritional Risk Screening 2002 [NRS2002] score), comorbid conditions (anemia, diabetes mellitus, hypothyroidism, hypertension, hyperlipidemia, chronic obstructive pulmonary disease, coronary artery disease, and prior surgical history), previous oncological therapies (chemotherapy and radiotherapy), tumour-related features (T stage, N stage, perineural invasion, tumour size, and tumour number), and preoperative laboratory indices (serum albumin, carcinoembryonic antigen [CEA], carbohydrate antigen 19–9 [CA19-9], and neutrophil-to-lymphocyte ratio [NLR]). Intraoperative variables included surgical approach, emergency procedure status, operative duration, estimated blood loss, intraoperative blood transfusion, and peripheral oxygen saturation (SpO_2_) levels. Postoperative variables were collected within 48 h after surgery and comprised laboratory biomarkers, including procalcitonin (PCT), C-reactive protein (CRP), and serum amyloid A (SAA), together with routine urinalysis findings, specifically the presence of proteinuria and haematuria.

### 2.3 Diagnosis of postoperative AKI and determination of associated factors

Postoperative AKI was defined according to the Kidney Disease: Improving Global Outcomes (KDIGO) criteria. AKI was diagnosed when any of the following conditions were met: (1) an absolute increase in serum creatinine of ≥0.3 mg/dL (≥26.5 μmol/L) from the preoperative baseline within 48 h after surgery; (2) a rise in serum creatinine to ≥1.5-fold above the preoperative baseline within 7 days postoperatively; or (3) a reduction in urine output to <0.5 mL/kg/h persisting for at least 6 h after surgery (23–25).

### 2.4 Development and evaluation of predictive models for machine learning algorithms

#### 2.4.1 Clinical prediction model development and evaluation were conducted using SPSS and R software

##### 2.4.1.1 Data preprocessing and dataset partitioning

Prior to model construction, comprehensive data preprocessing was performed. Clinical data from patients with colon cancer treated at Wuxi People’s Hospital, affiliated with Nanjing Medical University, between January 2010 and January 2020 were designated as the internal dataset, whereas contemporaneous data from Wuxi Second People’s Hospital, Tengzhou Central People’s Hospital, Tengzhou Hospital of Traditional Chinese Medicine, and Gaomi People’s Hospital constituted the external dataset. The internal dataset was used for model development, while the external dataset served as an independent cohort for external validation, enabling assessment of model generalizability and stability across institutions and patient populations. Within the internal dataset, patients were randomly allocated to a training set and a validation set in a 7:3 ratio using complete random sampling. The training set was used for model fitting and parameter learning, whereas the validation set was employed for hyperparameter optimization, mitigation of overfitting, and selection of the optimal model architecture. This strategy ensured sufficient sample size for robust training while preserving an adequately powered validation cohort for reliable evaluation of discriminative and calibration performance.

##### 2.4.1.2 Feature selection using statistical analysis and machine learning algorithms

In the second phase of model development, univariate and multivariate analyses were first performed within the internal dataset to screen for candidate predictors. Categorical variables were compared using the chi-square test. Normally distributed continuous variables were analysed with the independent-samples t test, whereas non-normally distributed variables were assessed using the rank-sum test. A two-sided P value <0.05 was considered statistically significant. To enhance the robustness and predictive relevance of feature selection, five machine learning algorithms—Extreme Gradient Boosting (XGBoost), Random Forest (RF), Support Vector Machine (SVM), Multilayer Perceptron (MLP), and k-nearest neighbours (KNN)—were subsequently applied to rank feature importance from a machine learning perspective. These algorithms were selected for their complementary strengths in feature evaluation, enabling identification of stable and informative predictors across diverse learning paradigms. XGBoost is a gradient-boosted tree model suitable for high-dimensional, nonlinear data, with feature importance assessed via gain. Random Forest evaluates feature contributions through ensembles of decision trees and is robust to noise and outliers. SVM constructs an optimal separating hyperplane, with importance derived from support vector weights or linear kernel coefficients, performing well in high-dimensional, small-sample settings. MLP captures complex nonlinear relationships, with feature relevance inferred from backpropagated weights. KNN assesses feature importance based on neighborhood classification and local data structure, identifying variables that shape local decision boundaries. By integrating traditional statistical analyses, which ensure clinical plausibility and inferential support, with machine learning–derived importance rankings that capture nonlinearities and interactions, we selected the intersection of statistically significant variables and consistently influential machine learning features as the final feature set for model construction.

##### 2.4.1.3 Model performance evaluation and validation

Predictive performance was comprehensively assessed across five domains: discrimination, calibration, clinical utility, internal generalization, and external transferability. Discrimination was evaluated using receiver operating characteristic (ROC) curves and the area under the curve (AUC), reflecting the model’s ability to distinguish patients with and without postoperative AKI, with higher AUC values indicating superior performance. Calibration was examined using calibration plots comparing predicted and observed risks, complemented by calculation of the Brier score to quantify overall prediction error and the accuracy of individualized risk estimates. Clinical utility was assessed through decision curve analysis (DCA), which estimates net benefit across a range of threshold probabilities while accounting for the clinical consequences of false-positive and false-negative predictions. This approach determines whether a model offers tangible benefit in guiding perioperative management, such as intensified monitoring, goal-directed fluid therapy, or implementation of renoprotective strategies. To evaluate internal generalization and model stability, k-fold cross-validation was applied to all machine learning algorithms within the internal dataset. The dataset was randomly partitioned into k approximately equal, non-overlapping subsets; in each iteration, one subset served as the validation set and the remaining k−1 subsets as the training set. This process was repeated k times, allowing each subset to function as the validation set once. Performance metrics, including AUC, accuracy, sensitivity, and specificity, were averaged across folds, with corresponding standard deviations calculated to assess overall performance and robustness. Compared with a single train–validation split, k-fold cross-validation improves data utilization, reduces partition-induced variability, facilitates detection of overfitting or underfitting, and yields more reliable estimates of real-world performance. Finally, external validation was conducted using data from Wuxi Second People’s Hospital, Tengzhou Central People’s Hospital, Tengzhou Hospital of Traditional Chinese Medicine, and Gaomi People’s Hospital to evaluate the model’s reproducibility and generalizability in independent clinical settings. The receiver operating characteristic area under the curve (ROC-AUC), DCA, and calibration curves were calculated to comprehensively assess predictive performance across different institutions and patient populations, thereby determining the model’s potential for cross-centre clinical implementation.

##### 2.4.1.4 Model robustness and overfitting assessment

This study also constructed Kolmogorov–Smirnov (KS) curves to quantify the maximum separation between the cumulative distribution functions of predicted probabilities for AKI and non-AKI patients. The KS statistic reflects the greatest vertical distance between the two distributions across all possible thresholds, providing an intuitive measure of class separation. In addition, parallel coordinates plots, learning curves, and confusion matrices for both the training and validation sets were generated to further examine model stability and potential overfitting. Parallel coordinates plots were used to visualize feature-level prediction patterns across samples, allowing the identification of extreme or inconsistent trajectories that might indicate over-adaptation to specific observations. Learning curves were constructed to assess the evolution of performance in the training and validation sets as a function of sample size; a widening gap between the two curves suggests overfitting, whereas convergence indicates good generalization. Confusion matrices were employed to compare classification behavior across datasets, enabling direct evaluation of whether near-perfect classification in the training set deteriorated substantially in the validation set. Collectively, these supplementary analyses provide a structured framework to detect over-optimization and assess model robustness beyond conventional discrimination metrics.

(5) Model interpretability: To enhance transparency and clinical relevance, SHAP analysis was applied at both global and individual levels. SHAP decomposes predictions into additive contributions from each feature, with summary plots showing overall feature importance and effect direction, dependence plots illustrating key feature trajectories and interactions, and force or waterfall plots demonstrating how features collectively influence predictions for individual patients. This framework systematically identified core risk factors, clarified their direction and magnitude, and captured interindividual heterogeneity, thereby improving model interpretability and clinical applicability.

## Results

### Baseline clinical characteristics and identification of risk factors for postoperative acute kidney injury

**Figure 1:**
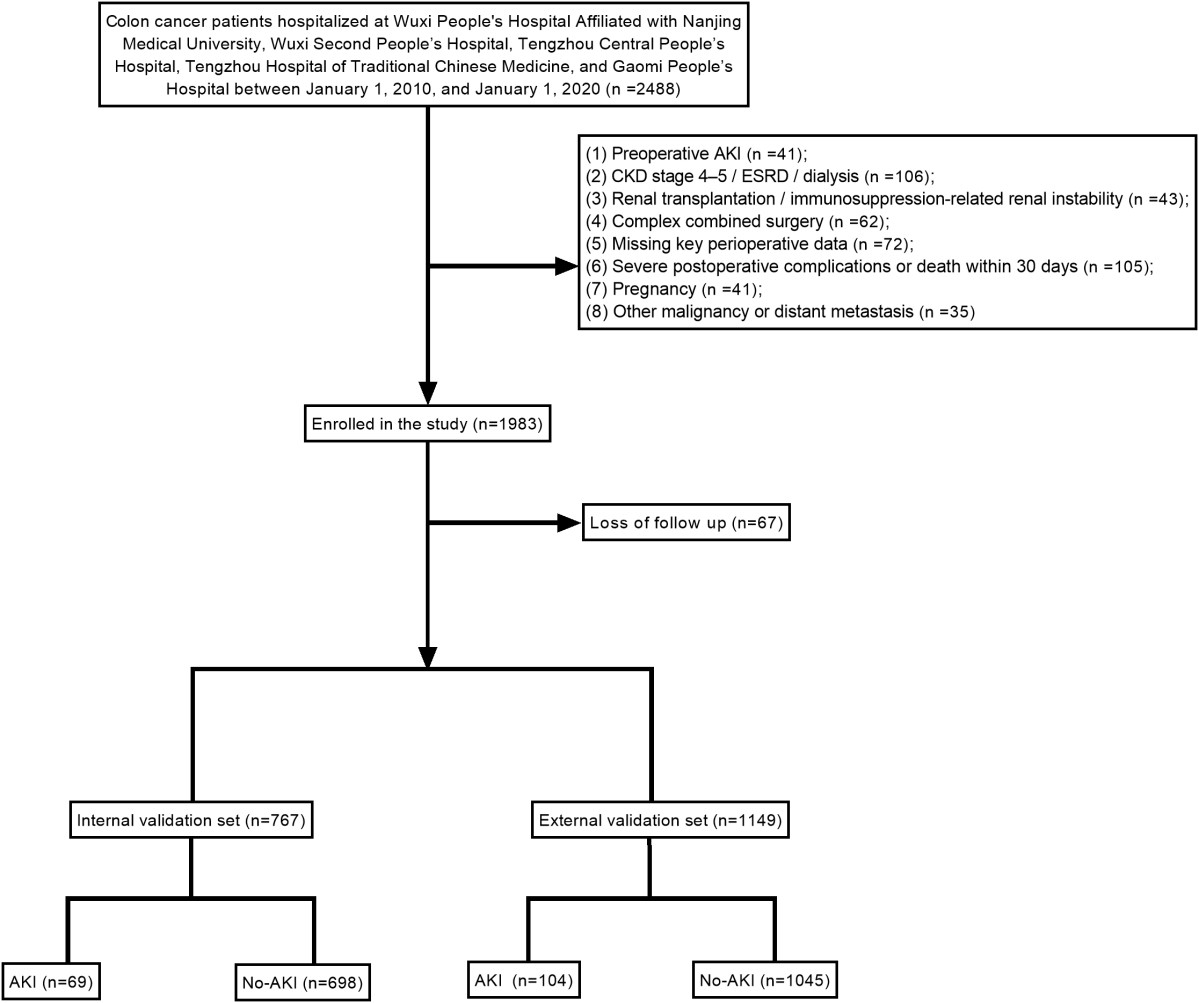
Flow diagram of patient selection for the postoperative AKI study.

A total of 1,916 patients with colon cancer were included in this study. Of these, 767 patients formed the internal cohort, among whom 69 developed postoperative AKI, whereas 1,149 patients constituted the external cohort, including 104 cases of AKI (**Figure 1**, **Table 1**).

**Table 1:** Baseline characteristics of variables in the internal datasets.

Variables		All (n=767)	Non-AKI (n=698)	AKI (n=69)	P-value
Sex	Female	352 (45.893)	319 (45.702)	33 (47.826)	0.736
	Male	415 (54.107)	379 (54.298)	36 (52.174)	
Age	<65	586 (76.402)	555 (79.513)	31 (44.928)	<0.001
	≥65	181 (23.598)	143 (20.487)	38 (55.072)	
BMI	<25 kg/m^2^	509 (66.362)	473 (67.765)	36 (52.174)	0.009
	≥25 kg/m^2^	258 (33.638)	225 (32.235)	33 (47.826)	
ASA	<3	502 (65.450)	460 (65.903)	42 (60.870)	0.402
	≥3	265 (34.550)	238 (34.097)	27 (39.130)	
Drinking history	No	528 (68.840)	479 (68.625)	49 (71.014)	0.683
	Yes	239 (31.160)	219 (31.375)	20 (28.986)	
Smoking history	No	526 (68.579)	489 (70.057)	37 (53.623)	0.005
	Yes	241 (31.421)	209 (29.943)	32 (46.377)	
ALB	≥30g/L	557 (72.621)	530 (75.931)	27 (39.130)	<0.001
	<30g/L	210 (27.379)	168 (24.069)	42 (60.870)	
NRS2002 score	<3	526 (68.579)	480 (68.768)	46 (66.667)	0.72
	≥3	241 (31.421)	218 (31.232)	23 (33.333)	
Surgical history	No	614 (80.052)	565 (80.946)	49 (71.014)	0.049
	Yes	153 (19.948)	133 (19.054)	20 (28.986)	
Anemia	No	567 (73.924)	536 (76.791)	31 (44.928)	<0.001
	Yes	200 (26.076)	162 (23.209)	38 (55.072)	
Hyperlipidemia	No	626 (81.617)	576 (82.521)	50 (72.464)	0.04
	Yes	141 (18.383)	122 (17.479)	19 (27.536)	
Hypertension	No	532 (69.361)	485 (69.484)	47 (68.116)	0.814
	Yes	235 (30.639)	213 (30.516)	22 (31.884)	
Diabetes	No	633 (82.529)	601 (86.103)	32 (46.377)	<0.001
	Yes	134 (17.471)	97 (13.897)	37 (53.623)	
Hypothyroidism	No	570 (74.316)	542 (77.650)	28 (40.580)	<0.001
	Yes	197 (25.684)	156 (22.350)	41 (59.420)	
COPD	No	646 (84.224)	590 (84.527)	56 (81.159)	0.464
	Yes	121 (15.776)	108 (15.473)	13 (18.841)	
CHD	No	669 (87.223)	608 (87.106)	61 (88.406)	0.758
	Yes	98 (12.777)	90 (12.894)	8 (11.594)	
Adjuvant Radiotherapy	No	652 (85.007)	600 (85.960)	52 (75.362)	0.019
	Yes	115 (14.993)	98 (14.040)	17 (24.638)	
Adjuvant Chemotherapy	No	623 (81.226)	570 (81.662)	53 (76.812)	0.325
	Yes	144 (18.774)	128 (18.338)	16 (23.188)	
Surgical procedure	Laparoscopic surgery	602 (78.488)	574 (82.235)	28 (40.580)	<0.001
	Open surgery	165 (21.512)	124 (17.765)	41 (59.420)	
Emergency surgery	No	564 (73.533)	517 (74.069)	47 (68.116)	0.285
	Yes	203 (26.467)	181 (25.931)	22 (31.884)	
Surgery time	<270 min	589 (76.793)	557 (79.799)	32 (46.377)	<0.001
	≥270 min	178 (23.207)	141 (20.201)	37 (53.623)	
Intraoperative bleeding	<100 ml	607 (79.140)	582 (83.381)	25 (36.232)	<0.001
	≥100 ml	160 (20.860)	116 (16.619)	44 (63.768)	
Blood transfusion	No	672 (87.614)	627 (89.828)	45 (65.217)	<0.001
	Yes	95 (12.386)	71 (10.172)	24 (34.783)	
SPO_2_	≥90%	624 (81.356)	591 (84.670)	33 (47.826)	<0.001
	<90%	143 (18.644)	107 (15.330)	36 (52.174)	
T-stage	T1~T2	545 (71.056)	515 (73.782)	30 (43.478)	<0.001
	T3~T4	222 (28.944)	183 (26.218)	39 (56.522)	
N-stage	N0	549 (71.578)	512 (73.352)	37 (53.623)	<0.001
	N1~N2	218 (28.422)	186 (26.648)	32 (46.377)	
PNI	No	697 (90.874)	634 (90.831)	63 (91.304)	0.896
	Yes	70 (9.126)	64 (9.169)	6 (8.696)	
Tumor number	<2	654 (85.267)	603 (86.390)	51 (73.913)	0.005
	≥2	113 (14.733)	95 (13.610)	18 (26.087)	
Tumor size	<5 cm	598 (77.966)	572 (81.948)	26 (37.681)	<0.001
	≥5 cm	169 (22.034)	126 (18.052)	43 (62.319)	
CEA level	<5 ng/ml	561 (73.142)	507 (72.636)	54 (78.261)	0.315
	≥5 ng/ml	206 (26.858)	191 (27.364)	15 (21.739)	
CA199 level	<37 U/mL	584 (76.141)	532 (76.218)	52 (75.362)	0.874
	≥37 U/mL	183 (23.859)	166 (23.782)	17 (24.638)	
PCT level	<0.05 ng/ml	595 (77.575)	537 (76.934)	58 (84.058)	0.176
	≥0.05 ng/ml	172 (22.425)	161 (23.066)	11 (15.942)	
CRP level	<10 mg/l	610 (79.531)	577 (82.665)	33 (47.826)	<0.001
	≥10 mg/l	157 (20.469)	121 (17.335)	36 (52.174)	
SAA level	<10 mg/l	540 (70.404)	501 (71.777)	39 (56.522)	0.008
	≥10 mg/l	227 (29.596)	197 (28.223)	30 (43.478)	
NLR	<3	540 (70.404)	514 (73.639)	26 (37.681)	<0.001
	≥3	227 (29.596)	184 (26.361)	43 (62.319)	
Proteinuria	No	636 (82.920)	576 (82.521)	60 (86.957)	0.35
	Yes	131 (17.080)	122 (17.479)	9 (13.043)	
Hematuria	No	660 (86.050)	602 (86.246)	58 (84.058)	0.617
	Yes	107 (13.950)	96 (13.754)	11 (15.942)	

OR, odds ratio; CI, confidence interval; BMI, body mass index; ASA, The American Society of Anesthesiologists; ALB, albumin; CA125, carbohydrate antigen 125; CA19-9, carbohydrate antigen 19-9; PCT, procalcitonin; CRP, C-reactive protein; SAA, serum amyloid A; NRS2002, nutrition risk screening 2002; CHD, coronary heart disease; COPD, chronic obstructive pulmonary disease; SPO_2_, percutaneous arterial oxygen saturation; AKI, acute kidney injury.

**Figure 2:**
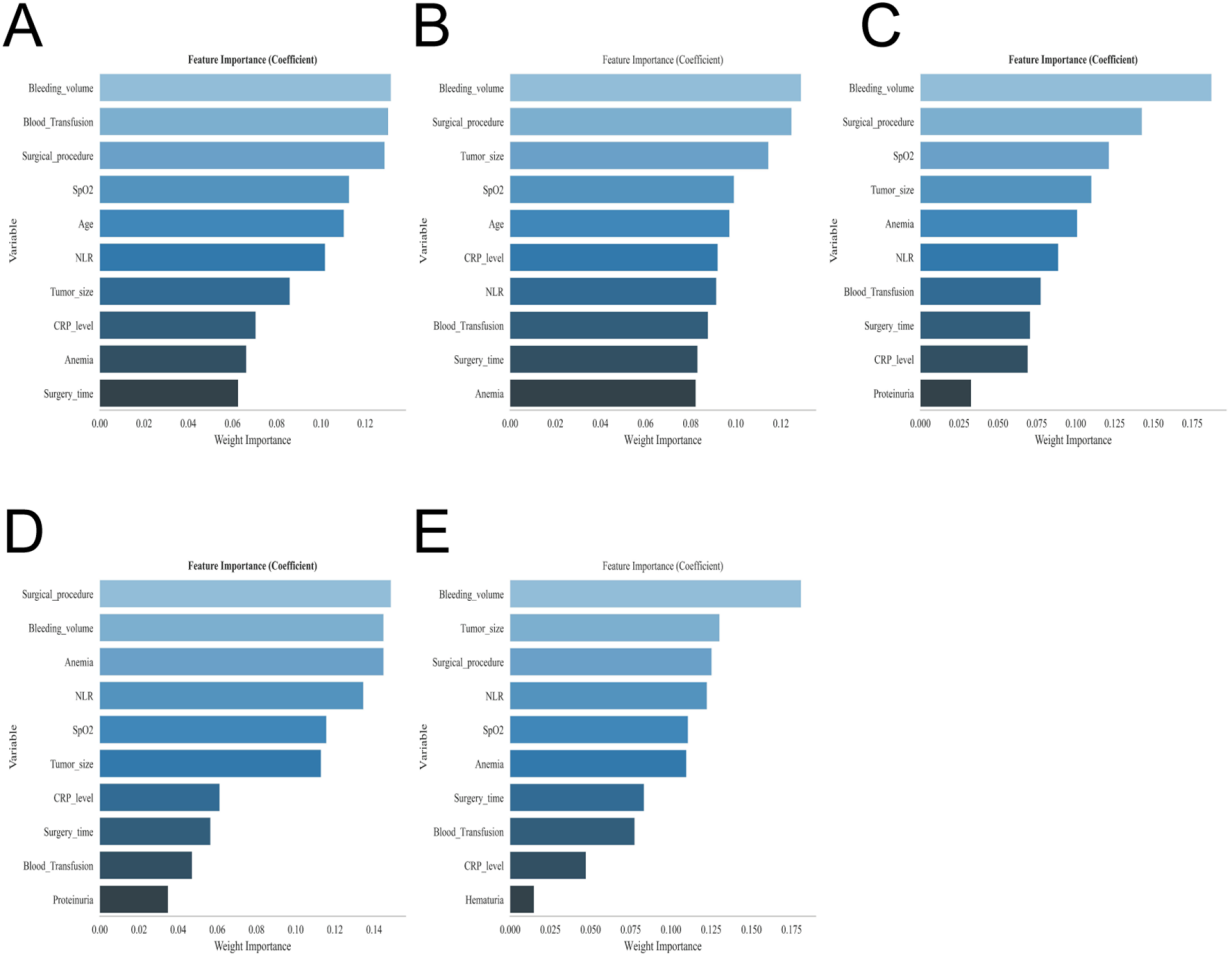
Variable importance ranking plots of the five machine learning models. **(A)** XGBoost variable importance ranking. **(B)** RF variable importance ranking. **(C)** SVM variable importance ranking. **(D)** KNN variable importance ranking. **(E)** MLP variable importance ranking.

Univariate and multivariate analyses revealed that advanced age, a history of anemia, hypoalbuminaemia, diabetes mellitus, hypothyroidism, surgical approach, operative duration, intraoperative blood loss, intraoperative hypoxaemia, blood transfusion, tumour size, tumour number, and elevated postoperative NLR and CRP levels were independently associated with postoperative AKI (all P < 0.05) (**Table 2**). Consistent with these findings, feature selection using XGBoost, RF, SVM, MLP, and KNN models repeatedly identified a history of anemia, surgical approach, operative duration, intraoperative blood loss, intraoperative hypoxaemia, perioperative blood transfusion, tumour size, and postoperative NLR and CRP levels as the most influential predictors of postoperative AKI (**Figures 2A–E**). Integrating conventional statistical analyses with machine learning–derived importance rankings, the final prediction model incorporated anemia, surgical approach, prolonged operative duration, increased intraoperative blood loss, intraoperative hypoxaemia, perioperative blood transfusion, tumour size, and postoperative NLR and CRP levels as key predictive variables. The original dataset utilized in this study is provided in **Supplementary Table 1**.

**Table 2:** Univariate and multivariate analysis of variables associated with postoperative AKI.

Variables		Univariate analysis		Multivariate analysis	
		OR, 95%CI	P-value	OR, 95%CI	P-value
Sex	Female	Reference			
	Male	0.918 [0.560,1.507]	0.736		
Age	<65	Reference		Reference	
	≥65	4.758 [2.861,7.912]	<0.001	10.737 [3.322,39.905]	<0.001
BMI	<25 kg/m^2^	Reference		Reference	
	≥25 kg/m^2^	1.927 [1.171,3.172]	0.01	1.531 [0.479,4.866]	0.466
ASA	<3	Reference			
	≥3	1.242 [0.747,2.065]	0.402		
Drinking history	No	Reference			
	Yes	0.893 [0.518,1.538]	0.683		
Smoking history	No	Reference		Reference	
	Yes	2.024 [1.227,3.337]	0.006	1.844 [0.548,6.140]	0.315
ALB	≥30g/L	Reference		Reference	
	<30g/L	4.907 [2.936,8.202]	<0.001	4.471 [1.351,15.994]	0.016
NRS2002 score	<3	Reference			
	≥3	1.101 [0.651,1.862]	0.72		
Surgical history	No	Reference			
	Yes	1.734 [0.997,3.015]	0.051		
Anemia	No	Reference		Reference	
	Yes	4.056 [2.446,6.726]	<0.001	13.548 [3.955,57.242]	<0.001
Hyperlipidemia	No	Reference		Reference	
	Yes	1.794 [1.022,3.151]	0.042	1.663 [0.404,6.525]	0.467
Hypertension	No	Reference			
	Yes	1.066 [0.627,1.813]	0.814		
Diabetes	No	Reference		Reference	
	Yes	7.164 [4.261,12.044]	<0.001	10.510 [2.979,43.173]	<0.001
Hypothyroidism	No	Reference		Reference	
	Yes	5.087 [3.048,8.493]	<0.001	8.893 [2.732,33.856]	0.001
COPD	No	Reference			
	Yes	1.268 [0.671,2.399]	0.465		
CHD	No	Reference			
	Yes	0.886 [0.410,1.913]	0.758		
Adjuvant Radiotherapy	No	Reference		Reference	
	Yes	2.002 [1.112,3.603]	0.021	1.790 [0.350,8.597]	0.471
Adjuvant Chemotherapy	No	Reference			
	Yes	1.344 [0.744,2.428]	0.326		
Surgical procedure	Laparoscopic surgery	Reference		Reference	
	Open surgery	6.778 [4.037,11.381]	<0.001	8.206 [2.528,30.591]	0.001
Emergency surgery	No	Reference			
	Yes	1.337 [0.784,2.280]	0.286		
Surgery time	<270 min	Reference		Reference	
	≥270 min	4.568 [2.748,7.591]	<0.001	4.777 [1.580,15.303]	0.006
Intraoperative bleeding	<100 ml	Reference		Reference	
	≥100 ml	8.83 [5.199,14.999]	<0.001	5.867 [1.779,21.412]	0.005
Blood transfusion	No	Reference		Reference	
	Yes	4.71 [2.710,8.187]	<0.001	8.164 [1.930,37.289]	0.005
SPO_2_	≥90%	Reference		Reference	
	<90%	6.025 [3.599,10.087]	<0.001	10.477 [3.141,42.391]	<0.001
T-stage	T1~T2	Reference		Reference	
	T3~T4	3.658 [2.208,6.062]	<0.001	0.664 [0.180,2.245]	0.52
N-stage	N0	Reference		Reference	
	N1~N2	2.381 [1.441,3.933]	0.001	1.399 [0.437,4.379]	0.563
PNI	No	Reference			
	Yes	0.943 [0.393,2.265]	0.896		
Tumor number	<2	Reference		Reference	
	≥2	2.24 [1.255,3.998]	0.006	10.520 [2.283,55.104]	0.003
Tumor size	<5 cm	Reference		Reference	
	≥5 cm	7.508 [4.447,12.676]	<0.001	35.746 [9.759,174.092]	<0.001
CEA level	<5 ng/ml	Reference			
	≥5 ng/ml	0.737 [0.406,1.338]	0.316		
CA199 level	<37 U/mL	Reference			
	≥37 U/mL	1.048 [0.590,1.861]	0.874		
PCT level	<0.05 ng/ml	Reference			
	≥0.05 ng/ml	0.633 [0.324,1.234]	0.179		
CRP level	<10 mg/l	Reference		Reference	
	≥10 mg/l	5.202 [3.119,8.675]	<0.001	5.391 [1.616,19.700]	0.008
SAA level	<10 mg/l	Reference		Reference	
	≥10 mg/l	1.956 [1.182,3.237]	0.009	0.608 [0.187,1.848]	0.39
NLR	<3	Reference		Reference	
	≥3	4.62 [2.760,7.734]	<0.001	6.959 [2.200,25.686]	0.002
Proteinuria	No	Reference			
	Yes	0.708 [0.342,1.466]	0.352		
Hematuria	No	Reference			
	Yes	1.189 [0.603,2.347]	0.617		

OR, odds ratio; CI, confidence interval; BMI, body mass index; ASA, The American Society of Anesthesiologists; ALB, albumin; CA125, carbohydrate antigen 125; CA19-9, carbohydrate antigen 19-9; PCT, procalcitonin; CRP, C-reactive protein; SAA, serum amyloid A; NRS2002, nutrition risk screening 2002; CHD, coronary heart disease; COPD, chronic obstructive pulmonary disease; SPO_2_, percutaneous arterial oxygen saturation; AKI, acute kidney injury.

### Model building and evaluation

**Figure 3:**
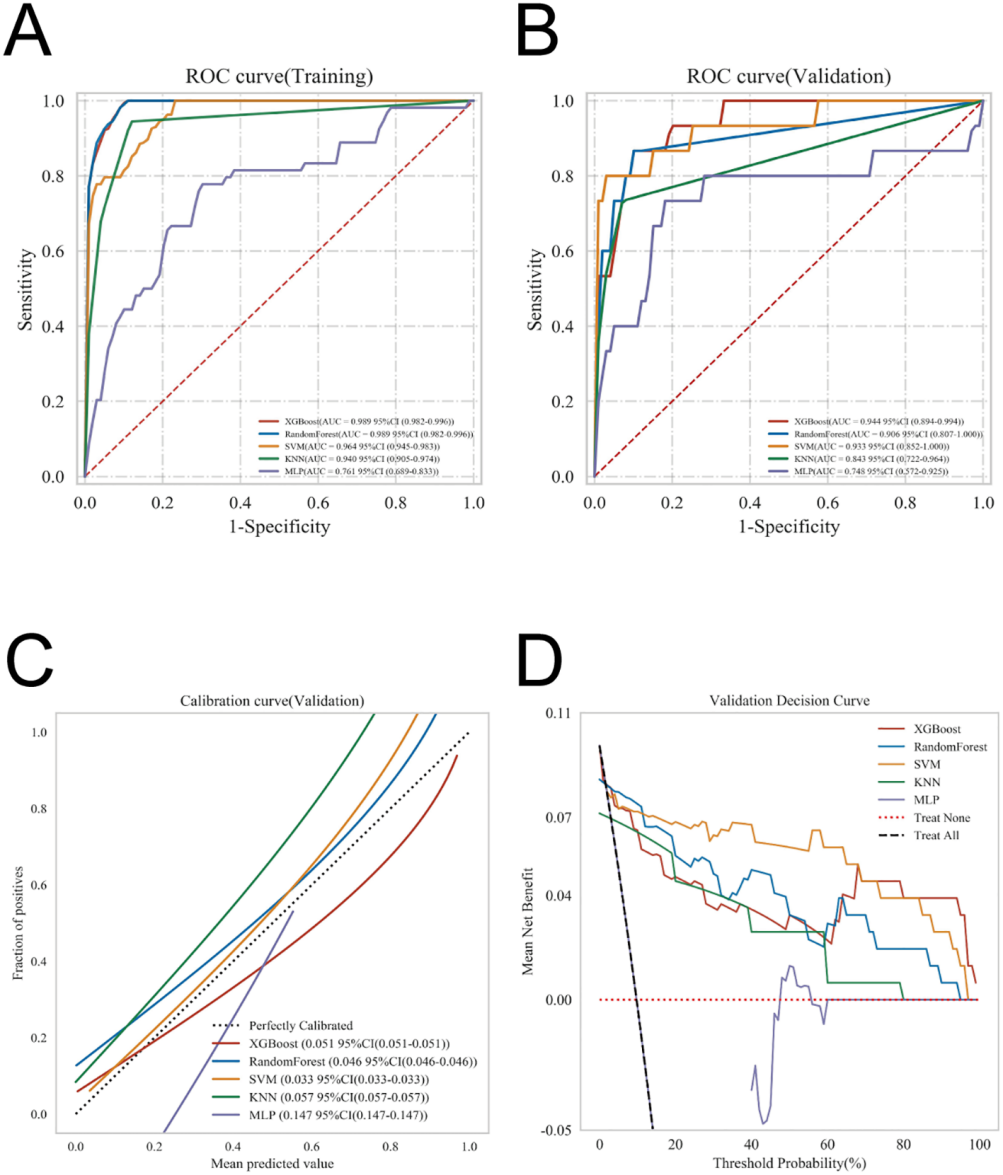
Model performance for predicting postoperative AKI. **(A)** ROC curves of the training set for the five models. **(B)** ROC curves of the validation set for the five models. **(C)** Calibration plots of the five models. The 45° dotted line indicates a perfect match between observed (y-axis) and predicted (x-axis) AKI probabilities. **(D)** DCA curves of the five models. The net benefit range is defined by the intersection of the model curve with the “All” and “None” curves.

ROC curve analysis showed that the XGBoost model achieved an AUC of 0.995 in the training set and 0.948 in the validation set, representing the highest discriminative performance among the five evaluated models (**Table 3**). Calibration curves for all models closely aligned with the ideal reference line, indicating excellent agreement between predicted risks and observed outcomes. DCA further demonstrated that each model conferred a positive net clinical benefit across a broad range of threshold probabilities, compared with strategies of treating all patients or treating none (**Figures 3A–D**).

**Table 3:** Performance evaluation of the five machine learning models for predicting postoperative AKI.

		AUC(95%CI)	Accuracy(95%CI)	Sensitivity(95%CI)	Specificity(95%CI)	F1 score(95%CI)	Kappa(95%CI)
KNN	training set	0.940 (0.905-0.974)	0.887 (0.871-0.893)	0.944 (0.924-0.954)	0.882 (0.872-0.892)	0.596 (0.552-0.621)	0.541 (0.522-0.564)
	validation set	0.843 (0.722-0.964)	0.909 (0.902-0.951)	0.733 (0.702-0.788)	0.928 (0.918-0.931)	0.611 (0.601-0.651)	0.561 (0.511-0.632)
XGBoost	training set	0.989 (0.982-0.996)	0.904 (0.901-0.910)	0.743 (0.740-0.746)	0.894 (0.890-0.899)	0.647 (0.637-0.667)	0.599 (0.589-0.621)
	validation set	0.944 (0.894-0.994)	0.916 (0.910-0.921)	0.733 (0.705-0.754)	0.935 (0.901-0.945)	0.629 (0.609-0.649)	0.582 (0.552-0.632)
RF	training set	0.989 (0.982-0.996)	0.902 (0.896-0.912)	0.871 (0.867-0.875)	0.893 (0.883-0.901)	0.643 (0.603-0.654)	0.594 (0.584-0.631)
	validation set	0.906 (0.807-1.000)	0.903 (0.900-0.953)	0.867 (0.862-0.878)	0.906 (0.902-0.916)	0.634 (0.614-0.644)	0.583 (0.563-0.601)
SVM	training set	0.964 (0.945-0.983)	0.791 (0.781-0.801)	0.867 (0.867-0.867)	0.771 (0.701-0.803)	0.458 (0.421-0.468)	0.372 (0.361-0.398)
	validation set	0.933 (0.852-1.000)	0.844 (0.804-0.857)	0.867 (0.827-0.888)	0.842 (0.802-0.888)	0.52 (0.512-0.542)	0.444 (0.424-0.465)
MLP	training set	0.761 (0.689-0.833)	0.71 (0.701-0.721)	0.778 (0.711-0.793)	0.703 (0.701-0.713)	0.321 (0.301-0.345)	0.21 (0.205-0.218)
	validation set	0.748 (0.572-0.925)	0.779 (0.759-0.799)	0.733 (0.711-0.783)	0.784 (0.764-0.799)	0.393 (0.343-0.412)	0.292 (0.282-0.312)

CI, confidence interval; KNN, k-nearest neighbor; XGBoost, extreme gradient boosting; RF, random forest; SVM, support vector machine; MLP, multilayer perceptron; AUC, area under the curve.

**Figure 4:**
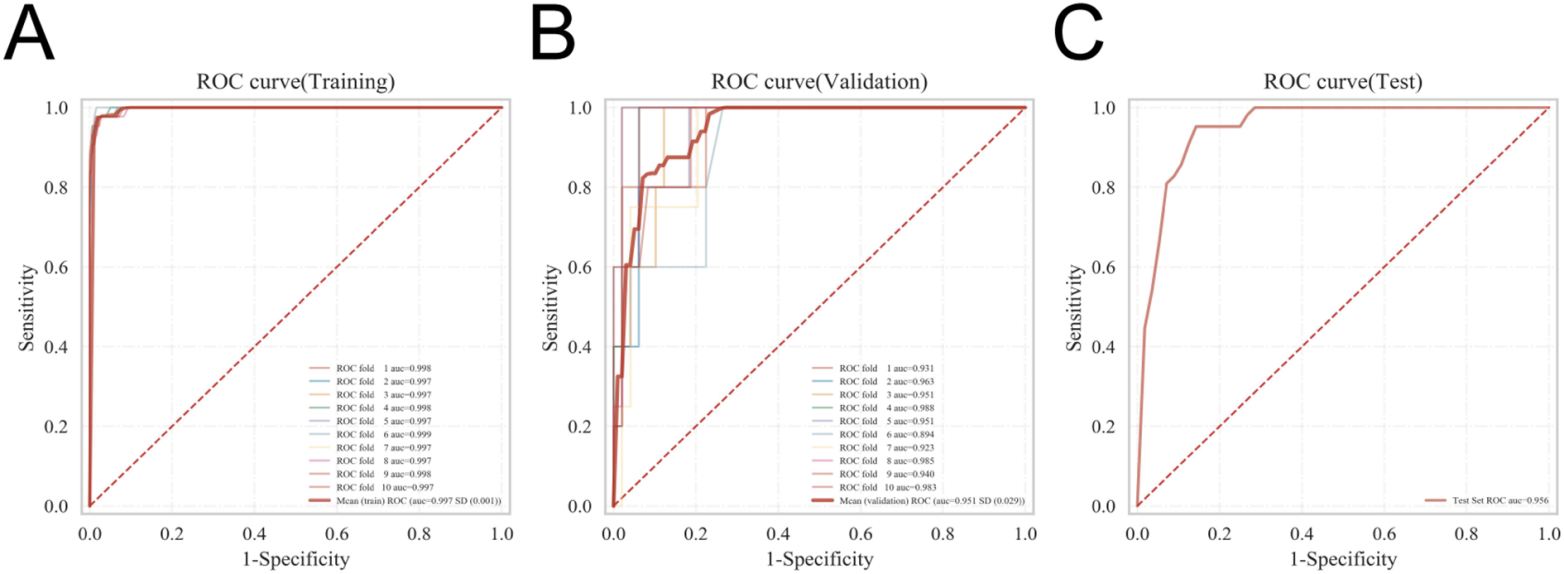
Validation of the XGBoost model. **(A)** ROC curve of the XGBoost model in the training set. **(B)** ROC curve of the XGBoost model in the validation set. **(C)** ROC curve of the XGBoost model in the internal test set.

Model generalizability was further assessed using k-fold cross-validation. From the internal dataset, 231 patients (30.12%) were randomly designated as the validation cohort, with the remaining patients used for training in a 10-fold cross-validation framework. Within the validation folds, the XGBoost model achieved an AUC of 0.9509 ± 0.0287, with a test-set AUC of 0.9561 and an overall accuracy of 0.9177 (**Figures 4A–C**). By comparison, the RF model yielded a validation AUC of 0.9279 ± 0.0536, a test-set AUC of 0.9151, and an accuracy of 0.9177. The SVM model achieved a validation AUC of 0.9044 ± 0.0372, a test-set AUC of 0.9052, and an accuracy of 0.9177. The KNN model demonstrated a validation AUC of 0.8574 ± 0.0748, a test-set AUC of 0.9036, and an accuracy of 0.8831, whereas the MLP model achieved a validation AUC of 0.8608 ± 0.0399, a test-set AUC of 0.8661, and an accuracy of 0.9134.

**Figure 5:**
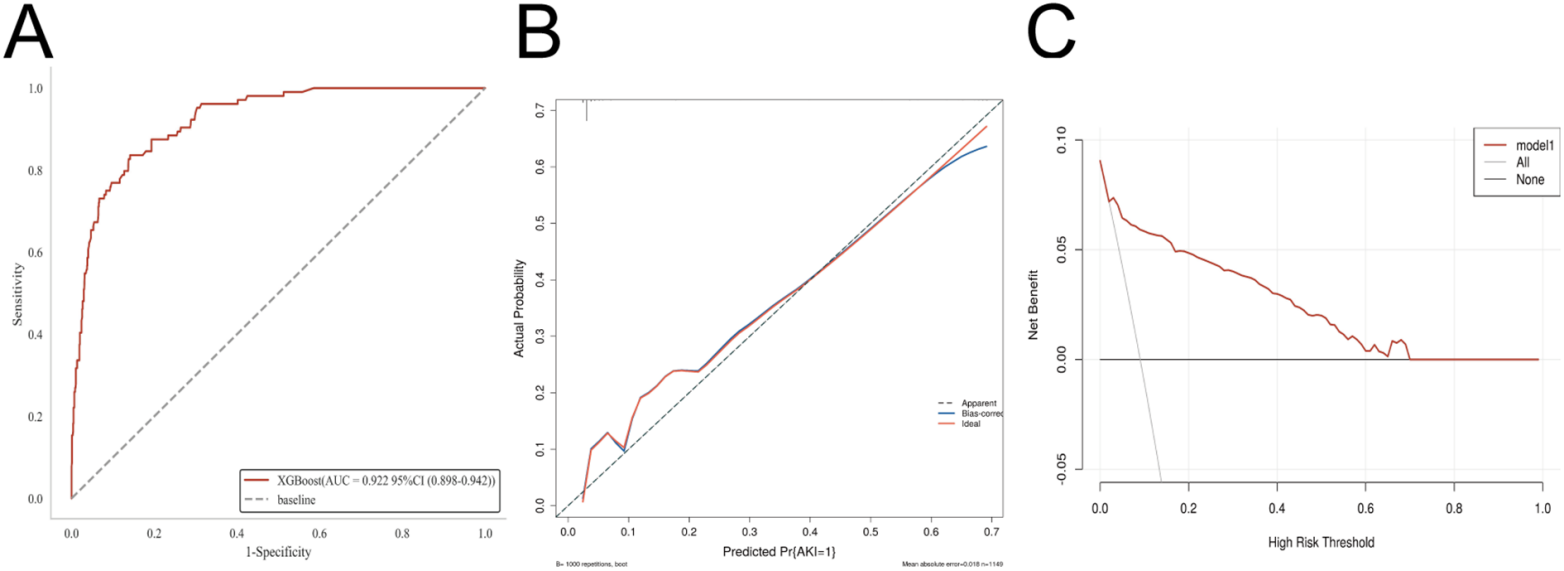
External validation of the model. **(A)** Receiver operating characteristic (ROC) curve demonstrating the discriminatory ability of the model in the external cohort. **(B)** Calibration curve illustrating the agreement between predicted and observed outcomes. **(C)** Decision curve analysis (DCA) assessing the clinical utility and net benefit of the model across a range of threshold probabilities.

On the basis of this comprehensive evaluation, XGBoost was selected for final model development. In the external validation cohort, the model achieved an AUC of 0.922 (95% CI, 0.898-0.942), demonstrating high accuracy and discriminative ability. DCA showed greater net benefit than “treat-all” or “treat-none” strategies, and the calibration curve closely aligned with the ideal line, indicating good agreement between predicted and observed outcomes. These results support the model’s strong generalizability and clinical applicability (**Figures 5A–C**).

**Figure 6:**
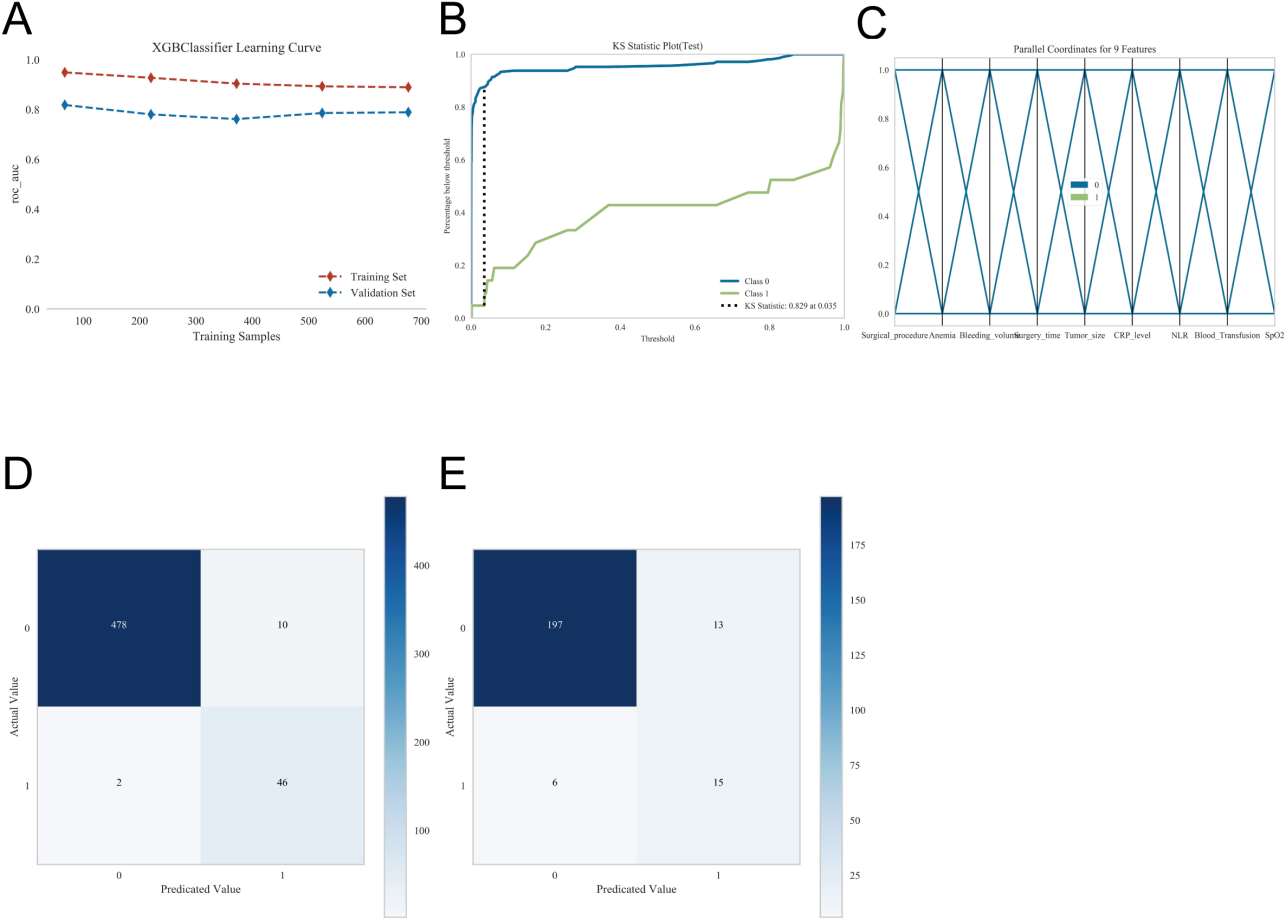
Model performance and evaluation metrics. **(A)** Learning curve depicting the model’s training progress and convergence. **(B)** Kolmogorov–Smirnov (KS) curve illustrating the separation between positive and negative cases. **(C)** Parallel coordinates plot visualizing the distribution of feature values across different outcome classes. **(D)** Confusion matrix for the training set, summarizing model predictions versus actual outcomes. **(E)** Confusion matrix for the validation set, summarizing model predictions versus actual outcomes.

The model demonstrated excellent performance across multiple evaluation metrics and visualizations. The learning curves showed that training and validation performance metrics converged closely and stabilized with increasing sample size, indicating minimal overfitting and robust generalization. The Kolmogorov-Smirnov (KS) curves revealed clear separation between the cumulative distributions of high-risk and low-risk samples, with a pronounced maximum vertical distance, reflecting strong discriminative ability. Parallel coordinates plots illustrated consistent feature contribution patterns across samples, highlighting distinguishable trends between high-risk and low-risk groups while maintaining stability without notable anomalies. Confusion matrices for both the training and validation sets further confirmed the model’s accuracy and robustness, with true positives and true negatives markedly exceeding false positives and false negatives. Collectively, these analyses demonstrate that the model maintains stable predictive behavior, effectively discriminates risk, and generalizes reliably across datasets (**Figures 6A–E**).

### Model explanation

**Figure 7:**
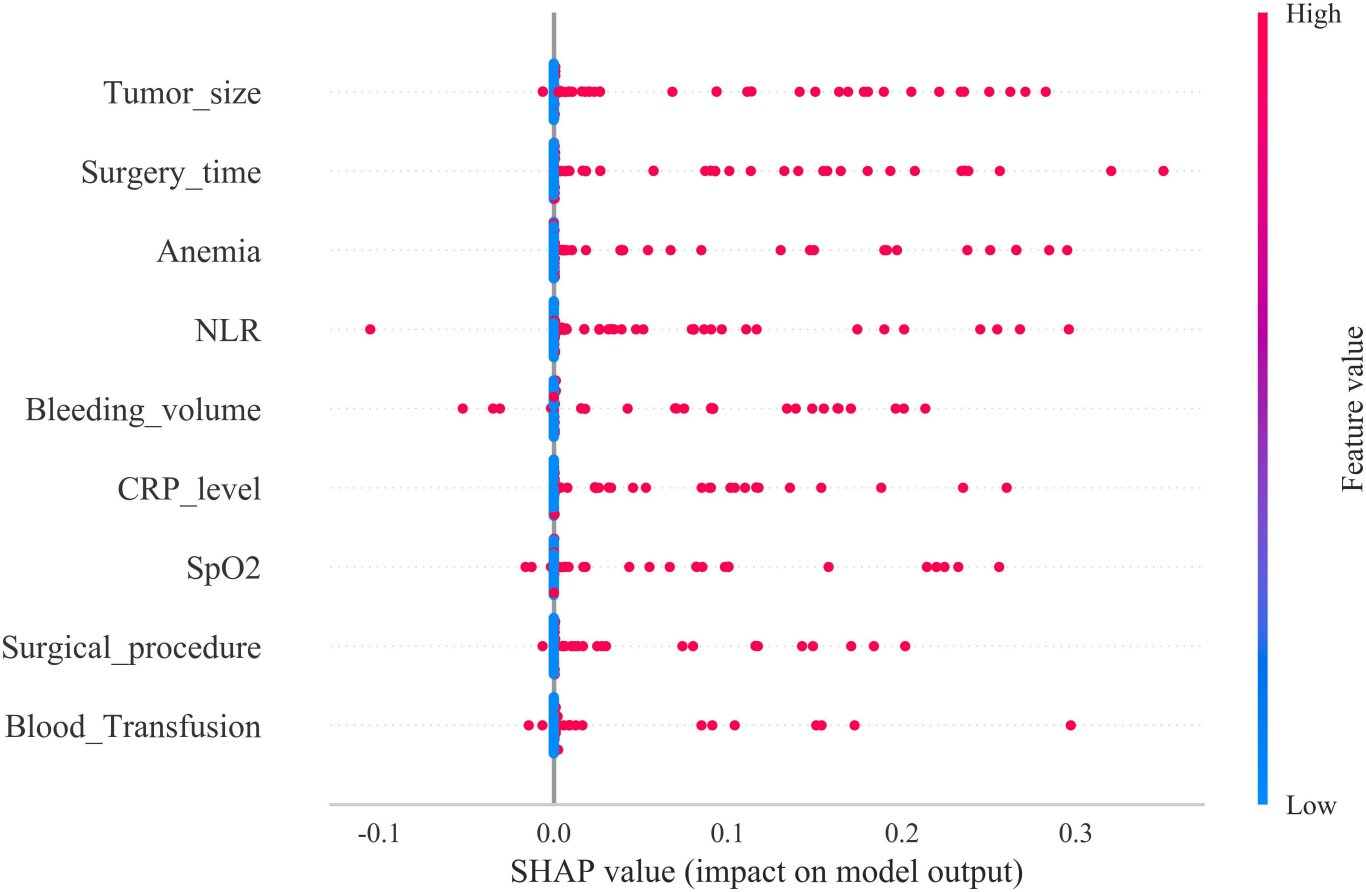
SHAP summary plot for postoperative AKI prediction. Risk factors are ordered along the y-axis according to the mean absolute Shapley values.

The SHAP summary plot revealed that the most influential determinants of postoperative AKI following complete mesocolic excision, ranked in descending order of importance, were tumour size, prolonged operative duration, anemia, elevated postoperative NLR, increased intraoperative blood loss, CRP level, intraoperative hypoxaemia, surgical approach, and intraoperative blood transfusion (**Figure 7**). In addition, SHAP force plots were generated to provide individualized explanations for five representative patients who developed AKI.

For Patient 1, the predicted probability of AKI was 0.92. The SHAP force plot indicated that multiple high-risk features exerted strong positive contributions, collectively shifting the prediction towards AKI occurrence. In particular, a history of anemia, intraoperative hypoxaemia, elevated NLR, and large tumour size yielded the largest positive Shapley values, underscoring their dominant roles in classifying this patient as being at extremely high risk.

For Patient 2, the predicted probability was 0.25, consistent with a relatively low-risk profile. Although a history of anemia, larger tumour size, and elevated NLR increased the predicted risk, a low postoperative CRP level contributed a pronounced negative Shapley value, counterbalancing these risk-enhancing features and ultimately shifting the prediction towards non-AKI, thereby maintaining a low overall probability.

For Patient 3, the predicted probability of AKI was 0.86, indicating a high-risk individual. This elevated risk was primarily driven by increased NLR, intraoperative hypoxaemia, larger tumour size, and prolonged operative duration, which collectively generated substantial positive Shapley values and pushed the prediction towards the AKI extreme.

For Patient 4, the predicted probability was only 0.04. Despite the presence of several established risk factors, including a history of anemia, intraoperative hypoxaemia, and an open surgical approach, the SHAP force plot demonstrated that their individual contributions were relatively modest. In contrast, multiple protective features—most notably lower postoperative inflammatory markers—exerted strong negative contributions, markedly attenuating the predicted risk and resulting in a very low final probability.

**Figure 8:**
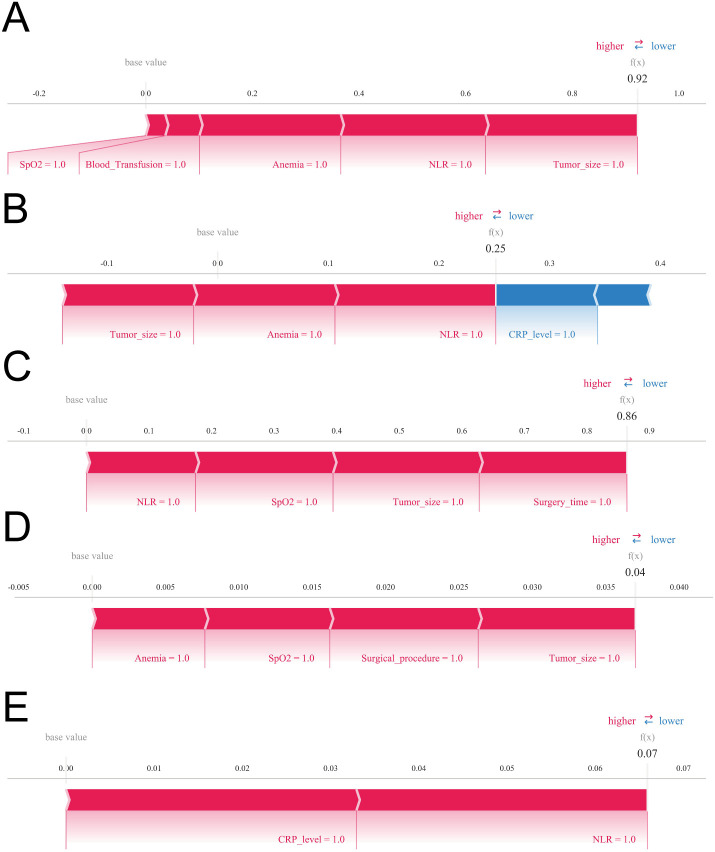
SHAP force plots illustrating individualized model explanations for postoperative AKI risk. Blue indicates features decreasing the predicted AKI probability (negative SHAP values), and red indicates features increasing the predicted probability (positive SHAP values). **(A)** Predictive analysis for Patient (I) **(B)** Predictive analysis for Patient II. **(C)** Predictive analysis for Patient III. **(D)** Predictive analysis for Patient IV. **(E)** Predictive analysis for Patient V.

For Patient 5, the predicted probability was 0.07. The modest risk was mainly attributable to elevated NLR and CRP levels; however, their overall contributions were limited. Negative contributions from other features predominated, leading the model to predict a low likelihood of postoperative AKI (**Figures 8A–E**).

## Discussion

In this study, we undertook a systematic comparison of five widely adopted machine learning algorithms—XGBoost, RF, SVM, KNN, and MLP—to predict AKI following CME. Although all five models achieved high predictive accuracy and demonstrated favourable clinical utility across ROC analysis, calibration assessment, and DCA, substantial heterogeneity in performance and methodological characteristics was evident. Among these approaches, the gradient boosting–based XGBoost model consistently exhibited superior performance. Specifically, XGBoost attained an AUC of 0.995 in the training cohort and preserved strong discriminative capacity in the validation cohort (AUC, 0.948). Its robustness was further corroborated by 10-fold cross-validation, yielding a mean validation AUC of 0.9509 ± 0.0287 and a test-set AUC of 0.9561, both exceeding those of the remaining models. This superiority is largely attributable to XGBoost’s capacity to model complex nonlinear relationships, capture high-order feature interactions, and attenuate overfitting through intrinsic regularisation, rendering it particularly well suited to structured, high-dimensional clinical datasets (12, 26). In addition, XGBoost provides stable outputs, high computational efficiency, and refined interpretability via SHAP-based explanations, collectively conferring substantial advantages in this context.

By comparison, although Random Forest effectively accommodates nonlinear associations and achieved a relatively high validation AUC of 0.9279 ± 0.0536, its ensemble architecture is inherently more complex and less interpretable. Moreover, RF may be subject to a dilution effect when a limited number of highly informative features dominate prediction, resulting in marginally inferior overall performance relative to XGBoost (27, 28). SVM demonstrated reasonable performance in high-dimensional, small-sample settings (validation AUC, 0.9044 ± 0.0372); however, its sensitivity to hyperparameter tuning and limited ability to capture extensive nonlinear interactions constrained its effectiveness in datasets characterised by numerous clinical variables and complex distributions (12, 14). The KNN algorithm, which relies on distance-based similarity within feature space, proved particularly susceptible to instability with increasing sample size or imperfect feature scaling, leading to the weakest and most variable performance (validation AUC, 0.8574 ± 0.0748). Although MLP is theoretically capable of learning complex feature representations, its dependence on larger sample sizes and susceptibility to convergence toward local optima limited its performance in this moderately sized cohort, resulting in a validation AUC of 0.8608 ± 0.0399 (29, 30).

From a calibration perspective, all five models demonstrated close concordance between predicted and observed probabilities, with calibration curves approximating the ideal reference line. DCA further indicated that each model conferred a net clinical benefit compared with treat-all or treat-none strategies, underscoring the potential clinical value of machine learning–based approaches for perioperative AKI risk stratification. Nevertheless, when ROC performance, cross-validation stability, interpretability, and real-world clinical applicability were considered in aggregate, XGBoost consistently outperformed the alternative algorithms and was therefore selected as the final modelling strategy.

Leveraging multicentre clinical data, we subsequently developed an XGBoost-based prediction model for postoperative AKI following CME and identified several key risk factors, including tumour size, intraoperative variables (operative duration, intraoperative blood loss, intraoperative SpO_2_, and open surgical approach), anaemia, and postoperative inflammatory markers (NLR and CRP). From a pathophysiological perspective, these factors can be broadly categorised into three interrelated domains—tumour burden, intraoperative stress with impaired oxygen delivery, and postoperative inflammatory response—which collectively drive the initiation and progression of AKI.

To enhance model transparency, SHAP was employed to quantify the contribution of individual features to AKI prediction. SHAP importance ranking identified tumour size as the most influential predictor, with a contribution substantially exceeding that of other clinical variables. This observation is both clinically and biologically plausible. Larger tumours often signify more advanced disease, frequently accompanied by chronic systemic inflammation, metabolic consumption, and malnutrition, thereby diminishing physiological reserve and tolerance to perioperative stress (31–33). Moreover, increased tumour burden is closely linked to greater surgical complexity, including prolonged operative time, extensive tissue manipulation, and heightened risk of intraoperative haemodynamic instability, collectively predisposing the kidneys to hypoperfusion and ischaemic injury. Larger tumours are also commonly associated with preoperative anaemia, hypoalbuminaemia, and elevated inflammatory markers, further exacerbating renal vulnerability during surgical stress.

At the individual level, SHAP force plots generated for representative patients consistently demonstrated that intraoperative haemodynamic instability, impaired oxygen delivery, and anaemia were dominant drivers of increased AKI risk. In this cohort, operative duration, intraoperative blood loss, intraoperative SpO_2_, and open surgical approach were all significantly associated with AKI. Prolonged surgery is typically accompanied by greater tissue trauma, sustained circulatory perturbations, and extended anaesthetic exposure, thereby prolonging renal ischaemic stress. Substantial blood loss reduces effective circulating volume and renal perfusion, while blood transfusion itself may provoke inflammatory activation and microcirculatory dysfunction (34–36). Reductions in intraoperative SpO_2_ reflect compromised systemic oxygen delivery; notably, the renal medulla is highly susceptible to hypoxia, and even transient desaturation may disrupt tubular energy metabolism and structural integrity (37–39). Anaemia, spanning both preoperative and intraoperative periods, represents a critical determinant of impaired renal oxygen transport, amplifying the deleterious effects of hypoperfusion. Collectively, these intraoperative factors converge on a central pathophysiological axis of hypoperfusion–hypoxia–microcirculatory dysfunction, which underpins AKI development.

Importantly, intraoperative blood transfusion emerged as an independent and highly contributory risk factor for AKI. SHAP analysis positioned transfusion prominently within the multivariable risk spectrum and demonstrated its direct positive contribution in high-risk individuals, suggesting effects beyond its role as a surrogate for bleeding severity. Mechanistically, transfusion may exacerbate renal injury through multiple overlapping pathways, including transfusion-related immunomodulation, storage lesion–associated oxidative stress, and circulatory overload or microthrombus formation, ultimately impairing renal perfusion and oxygenation (40–42). Residual inflammatory mediators in allogeneic blood products may further amplify inflammatory cascades shortly after transfusion, facilitating progression from reversible injury to sustained renal damage. Nevertheless, as an observational study, transfusion may function as both a causal exposure and a proxy for disease severity, introducing potential confounding (43–45). Although multivariable regression, machine learning–based feature selection, and SHAP interpretation helped isolate its independent signal, causality cannot be definitively inferred, warranting further investigation using advanced causal inference methodologies.

Another notable contribution of this study is the emphasis on postoperative inflammation as a key determinant of AKI progression. Postoperative NLR and CRP emerged as significant predictors, underscoring the role of systemic inflammation in sustaining and amplifying renal injury. Elevated NLR reflects neutrophilia and lymphopenia, indicative of heightened inflammatory activation and immune dysregulation, which can impair renal microcirculation and endothelial integrity (46–48). Elevated CRP further signals a pronounced inflammatory response, potentially driven by surgical trauma, ischaemia–reperfusion injury, or infection, thereby exacerbating tubular damage and promoting AKI persistence (49–51). By contrast with prior studies focused primarily on preoperative status or intraoperative haemodynamics, our findings highlight postoperative inflammation as a critical prognostic dimension.

In summary, postoperative AKI following CME appears to arise from the synergistic interplay of tumour burden, intraoperative hypoperfusion with impaired oxygen delivery, and postoperative inflammatory response. The primary objective of this study was not to develop a purely preoperative risk stratification tool, but rather to construct a comprehensive perioperative predictive model aimed at the early identification of patients at high risk for postoperative AKI. Therefore, in addition to preoperative variables, intraoperative and early postoperative parameters were incorporated to capture dynamic physiological changes that may critically influence renal outcomes. We acknowledge that the inclusion of postoperative variables does limit the model’s applicability for strictly preoperative decision-making. However, our intended clinical scenario focuses on early postoperative risk stratification and intensified monitoring rather than preoperative intervention planning. By integrating perioperative information, the model aims to enhance early identification of high-risk individuals, thereby facilitating closer surveillance, optimization of hemodynamic management, and timely implementation of nephroprotective strategies. Nevertheless, this study has several limitations. First, as a retrospective observational study, it may be subject to selection bias and does not allow for causal inference between predictors and AKI. Second, although we included preoperative, intraoperative, and early postoperative variables to optimize model performance, some potentially relevant factors—such as intraoperative exposure to nephrotoxic drugs, anesthesia depth, and fluid management strategies—were not captured, which may affect the comprehensiveness and robustness of the model. Finally, the model has not yet been prospectively evaluated in real-world clinical practice, and its utility in guiding clinical decision-making and improving patient outcomes remains to be established.

## Conclusion

Using multicenter clinical data, this study compared five machine learning algorithms for predicting AKI after CME and found that the XGBoost model demonstrated the best discriminative performance, stability, and generalizability. In addition, XGBoost provided robust interpretability through SHAP-based analysis, enabling transparent identification of key risk factors. The model highlighted tumor size, anemia, operative duration, intraoperative blood loss, SpO_2_, surgical approach, and postoperative inflammatory markers, including the neutrophil-to-lymphocyte ratio and C-reactive protein, as major contributors to AKI risk. These factors collectively influence AKI development through interconnected mechanisms involving tumor burden, intraoperative hypoperfusion and hypoxia, and postoperative inflammatory responses. This machine learning–based approach may facilitate early risk stratification and support targeted perioperative management to reduce the incidence of postoperative AKI after CME.

## Data Availability

The original contributions presented in the study are included in the article/**Supplementary Material**. Further inquiries can be directed to the corresponding author.

## Supplementary material

Supplementary Table 1 Raw Data.
